# Measles Serosurveys: A Solution in Search of the Right Problem

**DOI:** 10.3389/fpubh.2021.539325

**Published:** 2021-07-15

**Authors:** Archchun Ariyarajah, Natasha S. Crowcroft

**Affiliations:** ^1^Dalla Lana School of Public Health, University of Toronto, Toronto, ON, Canada; ^2^Centre for Vaccine Preventable Diseases, University of Toronto, Toronto, ON, Canada; ^3^Immunization, Vaccines and Biologicals, World Health Organization, Geneva, Switzerland

**Keywords:** measles, global health, serosurvey, decision making, low and middle income countries

In 2019, measles garnered the world's attention as the number of outbreaks significantly increased around the world. Over 860,000 cases were reported globally in 2019, which was more than double compared to the previous year and the highest on record since 1996 (1, 2). In Europe, Albania, the Czech Republic, Greece, and the United Kingdom lost their measles elimination status (3). It followed the disappointment when the World Health Organization (WHO) region for the Americas, the only region to have eliminated measles, lost its measles elimination status due to outbreaks in Venezuela in 2018 and Brazil in 2019 (2). This was a far cry from the optimism of 2012, when member states at the World Health Assembly endorsed the Global Vaccine Action Plan (GVAP) with the global target of measles elimination in at least five of the six WHO regions by 2020, which was not achieved (4). This failure to achieve global targets underscored how far ambition was out of step with the reality on the ground. In the new era, the Measles and Rubella Strategic Framework 2021-2030 aims to achieve and sustain regional measles elimination goals (5).

Major policy documents for immunization by leading global health organizations do not sufficiently discuss the utility and feasibility of serosurveys. Documents such as Gavi 5.0, the Immunization Agenda 2030, UNICEF Immunization Roadmap 2018-2030 do not mention serosurveys (6–8). Modeling conducted for the WHO Strategic Advisory Group of Experts (SAGE) Working Group on Measles and Rubella indicated that it was too soon to set an eradication target (9). In its latest review, the SAGE Working Group stated that the GVAP highlighted the critical role of good quality data to inform decision-making, including vaccine coverage and disease surveillance data; however, the role of serosurveys was not discussed (10). On the other hand, the WHO Measles Position Paper mentions the use of serosurveys along with other data sources to assess the accumulation of susceptible individuals in the population, to conduct follow-up campaigns, and to identify target age range for measles vaccination campaigns (11). The Measles and Rubella Strategic Framework 2021-2030 mentions the use of serosurveys to supplement routine data to validate administrative data, identify immunity gaps, and collect qualitative information on determinants of vaccination (5). WHO's Guidelines on the Use of Serosurveys in Support of Measles and Rubella Elimination highlights that serosurveys provide supplementary data, in addition to vaccine coverage and case surveillance data (12). However, it also emphasizes that financial costs, human resources, and logistics are challenges to conducting serosurveys and therefore, should only be undertaken if vaccination programs cannot be guided by other sources of information (12). Although serosurveys are mentioned in some of these major policy documents, there is no clear guidance on when or how they should be used.

Serosurveys are cross-sectional surveys that measure antibodies against a vaccine-preventable disease from a representative sample of a population to estimate immunity (13). Serosurveys can be used to measure measles-specific IgG or neutralizing antibodies. Measles-specific IgG antibodies bind to the measles virus but may require additional immune cells to neutralize the virus while neutralizing antibodies alone are sufficient to neutralize the virus. For measles, antibodies from previous infection or vaccination cannot be distinguished (14). Although vaccine coverage and disease surveillance data are used to infer immunity levels, these sources do not directly measure it (14). Poor data quality, such as overestimated coverage and under-reported cases, can lead to an overestimation of immunity and may miss immunity gaps within specific populations, which is evidenced in some low-income countries (15). In these situations, data from serosurveys can be used to complement data from other sources, including vaccine coverage and disease surveillance (16). Even with high quality data, only serosurveys can directly measure immunity, which would allow to detect phenomena such as waning immunity (17). Serosurveys may be most relevant to predict the risk of outbreaks and plan for targeted vaccination campaigns in the specific situation where longstanding immunity gaps are suspected but coverage data are not adequate to assess the risk because of quality issues, migration of populations, or gaps in specific age or other sub-groups in the population.

For measles, serosurveys have been used mainly in high-income settings to estimate burden of disease and determine whether herd immunity thresholds have been crossed, identify age groups and communities that lack immunity, and to evaluate impact of vaccination campaigns (17). However, serosurveys have many challenges that require clarity regarding when they are likely to be useful, particularly in low- and middle-income countries (LMICs). They are expensive, and it is often hard to source representative sera, and rare for those sera to be accompanied by the meta-data that are ideally needed, including immunization status. This requires countries to have the financial resources, logistical capacity, and opportunities to leverage other surveys that are representative of the population. Serosurveys measure antibody titers against measles, which rely on an agreed upon correlate of protection, the evidence for which remains weak (18). Additionally, they do not evaluate cell mediated immunity, which can provide protection even when antibodies are below a specified threshold (18, 19). Laboratory test methods are critical in determining the quality of the results. Generally, two serological tests are used: enzyme immune assays (EIAs) and virus neutralization assays. EIAs are cheap and do not require extensive training, and results can be obtained within a few hours (12). WHO recommends that laboratories use commercially-available EIAs that have an acceptable performance level (12). Conversely, neutralization assays are considered to be the gold standard because they measure neutralizing antibodies that prevent infection (12). However, these assays are expensive, require extensive training, and are not ideal for processing a large number of samples (12). Particularly in LMICs, serosurveys may be even more limited by available technology, lack of laboratory capacity, and limited financial ressources (17).

Measles elimination requires immunity to be ≥95%, which means that at least 95% of the population needs to be immune to prevent endemic tranmission (1). Extraordinarily high quality data are required to determine precisely whether the immunity gap is as small as <5%. Serosurveys could be a useful tool to resolve uncertainty and help identify when, where, and in whom immunity gaps are too large. This has been recognized in the WHO Regional Office for Europe published guidelines for members states for conducting serosurveys for measles and rubella elimination, which includes methods for sampling populations and laboratory testing (20). However, this document lacks a decision support tool to help countries identify when they should consider serosurveys, and it has not been adapted for other regions. WHO created a measles risk assessment tool for countries which contains a section that assesses the quality and sensitivity of surveillance data (21). A separate tool could be developed to help countries decide if a serosurvey is useful and feasible to estimate population immunity against measles. Factors to consider for this decision support tool includes the presence of a current outbreak, specific groups in the population that are thought to be at risk, such as migrant and displaced persons, and availability and quality of historical vaccination coverage and measles surveillance data. Finally, the absolute size of communities that are thought to be at risk is an important consideration. The critical community size to maintain measles transmission is less than half a million people (22). As cities and density of populations grow in size, the level of geographic granularity required of data to inform control of measles is shrinking well below the traditional levels of coverage and disease reporting, and may present an opportunity to explore the role of serosurveys. Additionally, it is important to consider remote rural areas that have shown to have lower vaccination coverage compared to urban areas, which poses a barrier to measles elimination (23).

Currently it remains unclear when exactly LMICs should consider using serosurveys to complement other data sources that infer population-level immunity (16). The high costs of conducting serosurveys remain a significant barrier. Although locally made EIA kits could be a potential solution, it may not be feasible in LMICs due to limited manufacturing and standard setting infrastructure. Cost-effectiveness studies of serosurveys are very limited. A modeling study found that measles vaccination campaigns triggered by serosurveys could be cost-effective but that in high incidence settings, vaccination campaigns triggered by an increase in cases may be more efficient (24). The WHO Regional Office for Europe recommends actions to reduce costs including using previously collected specimens and sampling in conjunction with household surveys like the Demographic and Health Survey (DHS) (20). In this case, the design of the original survey needs to be appropriate to answer questions about population immunity and adding blood sample collection may reduce acceptability of the original survey (16). Alternatively, serosurveys for various vaccine-preventable diseases, or other conditions, can be combined to reduce costs (20).

Overall, serosurveys can play an important role in identifying immunity gaps in populations, but the resources needed, and high costs require careful consideration. A decision tool based on the quality of surveillance and vaccine coverage data is needed to help countries decide whether and when serosurveys should be considered as a complementary data source to guide measles elimination efforts. A few case studies to demonstrate the utility (or otherwise) of measles serosurveys could be informative. Hopefully, strategies and lessons learned from conducting such studies in LMICs could be adapted for emerging infectious diseases to identify previously infected individuals and to better understand transmission dynamics. The widespread measles outbreaks in 2019 are a reminder that fresh ideas and approaches are needed if we wish the trajectory of measles control to get back on track.

## Author Contributions

AA and NC contributed to the conception of the manuscript. AA wrote the first draft of the manuscript. NC wrote sections of the manuscript. Both authors contributed to manuscript revision, read and approved the submitted version.

## Conflict of Interest

The authors declare that the research was conducted in the absence of any commercial or financial relationships that could be construed as a potential conflict of interest.
